# A 57-Year-Old Female Presenting With Cardiopulmonary Arrest Secondary to Severe Hypokalemia From a Fanconi-Like Syndrome: A Case Report

**DOI:** 10.7759/cureus.54659

**Published:** 2024-02-21

**Authors:** Christopher H Goss, Michael Robertson

**Affiliations:** 1 Internal Medicine, Charleston Area Medical Center, Charleston, USA

**Keywords:** refractory hypokalemia, general nephrology, fanconi, cardiac arrest, severe hypokalemia etiologies

## Abstract

Fanconi syndrome is a multi-factorial disorder that involves diffuse malfunction of the proximal convoluted tubule in the kidney. Renal wasting of potassium, glucose, bicarbonate, amino acids, and phosphorus characterize the condition. We report a case of a 57-year-old female who presented to our emergency department with cardiopulmonary arrest. After successful resuscitation, she had extensive workup to uncover the cause of her cardiac arrest. She had extensive negative workup but was found to have severely low potassium, prompting further evaluation. She was noted to have elevated urine potassium, with a trans-tubular potassium gradient of 9. She was also found to have severe glycosuria, hypophosphatemia, proteinuria, and an elevated urine anion gap, suggesting proximal convoluted tubular dysfunction. The hypokalemia noted on admission was thought to have been the causative factor for the cardiopulmonary arrest and was thought to be due to proximal tubule dysfunction, with the major suspected diagnosis being a Fanconi-like syndrome. This report highlights the diagnosis and treatment of hypokalemia, the broad differential involved with hypokalemia, and the syndromes involved with renal potassium wasting. This report also seeks to raise awareness of the association of renal potassium wasting with cardiopulmonary arrest.

## Introduction

Fanconi Syndrome is an uncommon and complex disorder characterized by a diffuse malfunction in the proximal renal tubules that impairs the reabsorption of essential substances including glucose, amino acids, phosphate, and bicarbonate [1,2]. Fanconi syndrome can be either genetic or acquired through other means [1-3]. Primary or hereditary Fanconi syndrome, often inherited in an autosomal recessive pattern, has been linked to specific gene mutations that influence transporters responsible for the reabsorption of essential substances from renal tubules [1,2]. Acquired Fanconi syndrome may result from exposure to medications including antiretrovirals and antibiotics, toxins, or medical conditions like multiple myeloma, cystinosis, or Wilson's disease which disrupt normal proximal renal tubule functioning [1,2].

Diagnosing Fanconi syndrome typically involves a combination of clinical evaluation, laboratory tests, and genetic analysis [2-4]. Patients may exhibit symptoms including excessive thirst and urination, muscle weakness, bone pain, and growth restriction in children [4]. Laboratory tests often show elevated urine concentrations of substances including glucose, amino acids, protein, phosphates, and bicarbonate [1,2]. Genetic testing can identify mutations linked to hereditary Fanconi syndrome while kidney biopsies may be conducted to assess tubular damage severity if kidney injury is present [1,2].

Management of Fanconi Syndrome depends on its underlying cause. For the acquired form, discontinuing offending medications or treating underlying medical conditions that contribute to the condition are crucial, as is correcting specific nutrient and electrolyte imbalances caused by excessive renal loss through nutrient supplementation and hydration [3-5]. With hereditary syndrome, treatment should focus on alleviating symptoms while preventing complications; regular monitoring and creating personalized plans of care should help ensure an improved overall quality of life [3-5].

Genetic analyses continue to uncover new mutations and further our knowledge of molecular mechanisms underlying this disorder [4]. Knowledge gained through research may lead to targeted therapies or gene-based interventions for both hereditary and acquired forms. Additionally, efforts are being undertaken to identify novel diagnostic markers and non-invasive monitoring techniques that will facilitate early diagnosis and management of this condition [4]. Emerging gene therapies hold hope of improving the quality of life for individuals living with Fanconi syndrome [1-4].

This case study features the diagnosis and management of hypokalemia, proximal convoluted tubule defects, and a suspected Fanconi-like syndrome in a 57-year-old female who presented with cardiopulmonary arrest and was found to have severe hypokalemia, glycosuria, proteinuria, hypophosphatemia and an elevated urine anion gap, indicative of proximal tubular dysfunction.

## Case presentation

**Figure 1:**
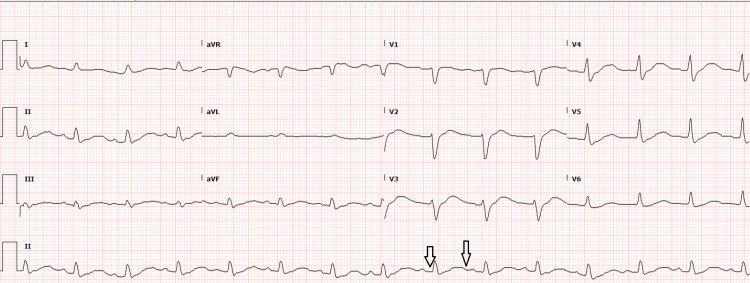
EKG 1: Electrocardiogram taken immediately after return of spontaneous circulation. Initial EKG with arrows showing a prolonged QT interval.

**Figure 2:**
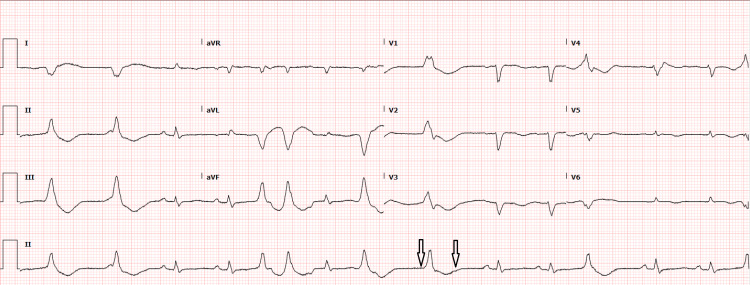
EKG 2: Electrocardiogram taken approximately four hours after return of spontaneous circulation. Subsequent EKG with arrows showing a shorter QT interval compared to previous EKG.

A 57-year-old female with a past medical history of hyperlipidemia, heart failure with reduced ejection fraction, depression, and hypertension presented to the emergency department secondary to experiencing a cardiac arrest at home. Prior to EMS arrival, she received CPR from her son. En route to the hospital, ROSC was achieved by paramedics. The initial EKG rhythm during the cardiac arrest was documented as ventricular fibrillation, but an actual copy of the EKG was not documented (Figure 1). The EKG after the return of spontaneous circulation showed multiple premature ventricular contractions, a left bundle branch block that appeared new when compared to previous EKGs, a PR interval of 166 ms, QRS duration of 129 ms, and QTc of 535 ms. In the emergency department, she was brought in already having been intubated, a left internal jugular triple-lumen central venous catheter was placed, and the ICU service was called for admission. During admission, the patient subsequently went into ventricular fibrillation again with the loss of pulse, underwent two additional rounds of CPR per ACLS protocol, was bolused and infused with amiodarone, and received one shock at 150 joules. On the stat critical care panel, which includes an arterial blood gas (ABG), basic metabolic panel, hemoglobin and hematocrit, and serum lactic acid level, she was found to have a critical potassium of 1.7, which was subsequently repleted. A total of 260 meq of potassium was administered during and after the arrest. Of note, she was not provided with bicarbonate or phosphorous during the code or throughout hospitalization. The first EKG (Figure 1) shows the initial rhythm after obtaining the return of spontaneous circulation, with prolonged QTc. The second EKG (Figure 2) shows a reduced QTc of 450 ms after potassium replacement.

A summary of admission labs is noted in Table 1. Epinephrine, calcium gluconate, glucose, and potassium were administered during the arrest. Return of spontaneous circulation was achieved after the second round of CPR. By the end of the first day of admission, serum potassium was 3.3. The potassium went up to 4.2 the next morning, then 3.2, and then 2.8, requiring additional potassium supplementation. Throughout her lengthy hospital course, the patient required daily oral potassium supplementation, around 40-80 meq per day. She was eventually placed on amiloride, a potassium-sparing diuretic, and sacubitril-valsartan to assist with maintaining the potassium levels.

**Table 1:** Serum and urine laboratory values on hospital admission BUN – blood urea nitrogen; mcL – microliters; g/dL – grams per deciliter; mmol/L – millimoles per liter; mg/dL – milligrams per deciliter; pg/mL – picograms per milliliter; ng/L – nanograms per milliliter

Lab Test	Admission Result	Reference Range
White Count	18.9	4.8-10.8 x 10^3/mcL
Hemoglobin	14.5	14-16 g/dL
Sodium	139	136-145 mmol/L
Potassium	1.7	3.5-5.1 mmol/L
Chloride	102	98-107 mmol/L
Bicarbonate	18	21-32 mmol/L
Glucose	121	74-106 mg/dL
Calcium	9.1	8.6-10.3 mg/dL
BUN	11	7-25 mg/dL
Creatinine	1.0	0.7-1.3 mg/dL
Magnesium	2.2	1.8-2.4 mg/dL
Phosphorous	1.1	2.5-4.9 mg/dL
Lactate	7.2	0.5-2.0 mmol/L
Serum Osmolality	294	
Urine Urea	390	
Urine Calcium	4.1	
Urine Chloride	156	
Urine Creatinine	66.5	
Urine Glucose	2204	
Urine Potassium	56.5	
Urine Sodium	104	
Urine Osmolality	703	
Urine phosphorus	22.6	
Urine Uric acid	25	
Urine protein	69.7	
Urine microalbumin	7.7	
Urine protein/creatinine ratio	1,667	

Point-of-care ultrasound revealed no evidence of any identifiable cardiac tamponade, and there was no evidence of pneumothorax on chest x-ray or ultrasound. CT of the head and cervical spine was negative for any acute process. CT angiogram of the chest for pulmonary embolism was negative. Other patient history was limited and obtained by her mother. It appeared that the patient had undergone a nuclear medicine cardiac stress test that was negative 10 months prior secondary to dyspnea on exertion. Her home medications included escitalopram, aspirin, and rosuvastatin. When more medically stable, she underwent left heart catheterization that showed nonobstructive coronary artery disease. She also underwent a cardiac MRI to look for infiltrative cardiomyopathy, and this was negative as well.

The hypokalemia noted on admission, appeared to be new onset hypokalemia, with no prior history of hypokalemia on outpatient or previous inpatient labs. There were no recent adjustments to home medications. There were no diuretics on the patient’s home medications, and no recent gastrointestinal symptoms of nausea, vomiting, or diarrhea prior to presentation which may have accounted for the hypokalemia. The urine electrolytes on admission, as noted in Table 1, were elevated. The urine anion gap was positive and the transtubular potassium gradient (TTKG) was calculated to be 9, indicating renal losses of potassium. Further workup of the hypokalemia included a renal artery duplex, which was negative for renal artery stenosis, a cortisol level that was within normal limits, and plasma aldosterone and renin levels which did not suggest primary hyperaldosteronism. An HIV test was negative, and the magnesium level on and throughout admission was within normal limits. Given no prior episode of hypokalemia, other possibilities of the new onset hypokalemia in this patient with cardiac arrest could have been secondary to the use of epinephrine boluses, but the patient had persistent hypokalemia days into hospitalization requiring daily supplementation. Finally, given the wide discrepancy between microalbuminuria and proteinuria, there was concern for non-albuminuric proteinuria, which could have been secondary to immunoglobulins. Thus, urine and serum protein electrophoreses were ordered due to concern for paraprotein-related kidney disease as a possible trigger for renal wasting. The results of these tests did not suggest any monoclonal gammopathy like monoclonal gammopathy of undetermined significance or multiple myeloma. Given elevated urine potassium, severe glycosuria despite relatively well-controlled serum glucose, hypophosphatemia, proteinuria, and elevated urine anion gap, this further pointed to the cause of renal wasting being a proximal convoluted tubule defect, including but not limited to Fanconi syndrome.

## Discussion

In this case, a patient presented to the emergency department with both cardiac arrest and severe hypokalemia. Her medical history included heart failure, hyperlipidemia, and hypertension - making the initial clinical picture more complicated. This patient’s cardiac arrest was an alarming event and its causes were fully investigated, as the differential for causes of cardiac arrest is broad. Many of the common causes for cardiac arrest were ruled out, making hypokalemia the most likely triggering factor for the arrest. For the hypokalemia, the absence of recent adjustments to blood pressure medications, previous history of hypokalemia, diuretic use, or gastrointestinal symptoms ruled out some common sources of hypokalemia, prompting further workup. The tubular potassium wasting, severe glycosuria, proteinuria, and hypophosphatemia pointed more toward proximal convoluted tubule dysfunction and Fanconi syndrome being the causative factors for the hypokalemia, and ultimately the cardiac arrest.

Diagnosing Fanconi syndrome, or a Fanconi-like syndrome as in this case, can be difficult due to its rarity and nonspecific symptoms that are easily overlooked. Therefore, this condition often necessitates a multidisciplinary approach, including the involvement of nephrologists, endocrinologists, and cardiologists in order to navigate the complex diagnostic process successfully. In this instance, nephrology was consulted, who assisted with the workup, and ultimately agreed that the most likely driving force for the hypokalemia was proximal convoluted tubule dysfunction. After a discussion with the primary team, the attending nephrologist started amiloride and sacubitril-valsartan to assist with reducing urinary losses of potassium. An attempt was made to collect 24-hour urine samples to quantify urine electrolytes, bicarbonate, uric acid, and phosphorus, but the patient was discharged to a skilled nursing facility prior to a full 24-hour urine collection.

Laboratory tests played an instrumental role in ascertaining tubular dysfunction in this patient. Analysis of urine electrolytes, with elevated potassium levels and positive urine anion gap, provided invaluable clues that suggest renal losses of potassium as well as dysfunction within proximal convoluted tubules. These findings emphasize the significance of taking a systematic approach to laboratory testing when diagnosing complex renal disorders. Such tests not only confirm the presence of disease including hypokalemia, but can also rule out other potential causes and lead clinicians toward making accurate diagnoses.

Long-term monitoring and care are integral parts of managing Fanconi syndrome. Patients need regular appointments with their healthcare provider to monitor electrolyte levels, kidney function, and overall health status - this allows for early detection of any deviations from treatment plans as well as timely adjustments if necessary [1,2]. Care plans tailored specifically for each patient are essential in meeting the unique needs of every individual, taking into account factors like age, severity of condition, and complications as well as education and support for patients and their families [1,2].

The evaluation of hypokalemia involves conducting an intensive and detailed process that assesses potential causes for low potassium levels in the blood. This is essential since hypokalemia can arise from renal and non-renal causes [5,6]. When applied to this patient's case specifically, hypokalemia testing led to the diagnosis of proximal tubular dysfunction. As noted with the patient in this case, a complete review of any medications, comorbidities, dietary habits, or recent gastrointestinal symptoms including vomiting and diarrhea must occur to ascertain any possible sources of potassium loss [6,7]. Furthermore, certain medications including loop and thiazide diuretics, and genetic conditions including Bartter and Gitelman syndrome, can lead to hypokalemia and thus must be considered in the differential [6-8].

Fanconi syndrome exhibits elevated urinary potassium levels, indicative of renal loss of potassium. To further elucidate renal vs GI losses, a TTKG can be calculated [6-8]. For example, this patient had a TTKG value of 9, suggesting renal potassium losses. Besides the serum and urine potassium levels and TTKG calculation, evaluation of hypokalemia involves other labs. ABG analysis can detect metabolic acidosis that may result from bicarbonate loss through urine excretion [8]. A basic metabolic panel measures electrolytes such as sodium, bicarbonate, and chloride to provide more insight into a patient’s acid-base balance [8]. This is especially important because, in the setting of metabolic acidosis, a transcellular shifting of potassium can occur [6-8]. Thus, a portion of the hypokalemia noted on admission may have been in the setting of the high anion gap acidosis, representing a marked depletion in total body potassium stores [6-8]. Hemoglobin and hematocrit tests help assess for anemia or blood loss which could contribute to hypokalemia [8]. Elevated serum lactic acid levels may indicate tissue hypoxia and may be especially significant in cases of severe hypokalemia and cardiac arrest, as was seen with this patient [6-8].

In certain instances, additional testing may be necessary. Imaging studies such as renal ultrasound or CT scans may be performed to assess kidney anatomy and identify structural abnormalities, such as unilateral renal artery stenosis, which can lead to chronic renin-angiotensin-aldosterone activation and hypokalemia [8]. Additional workup for hypokalemia can include ruling out primary hyperaldosteronism, a syndrome characterized by hypertension and hypokalemia [9]. Hormone testing including plasma aldosterone and renin levels should be conducted, with an elevated ratio suggesting the diagnosis [8-10]. An 8 am cortisol level should be measured to exclude Cushing syndrome [8-10]. A notable percentage of people with AIDS present with hypokalemia, thus HIV testing may also be warranted [8-10]. Finally, if there is cause for suspicion of paraprotein-related kidney disease, serum, and urine protein electrophoreses can assist in the detection of any abnormal proteins present in either serum or urine samples [8-10].

This report emphasized the importance of timely CPR as well as the assessment and replacement of potassium in patients with hypokalemia who present with cardiac arrest. When severe cardiac arrest or instability due to hypokalemia is recognized, advanced cardiac life support or other relevant resuscitation protocols should be implemented immediately [11]. In the case of severe hypokalemia, immediate potassium replacement may be life-saving, and central venous access is critical. It is important to closely monitor cardiac status while administering potassium. It is also important to adjust infusion rates according to ECG changes in cardiac monitoring [11,12]. In severe potassium fluctuations or cardiac arrest, additional infusions such as calcium and magnesium can also be beneficial [11,12].

Effective interventions require a multidisciplinary approach that includes emergency physicians, internists, nephrologists, and cardiologists, as well as clinical pharmacists. It is important to address and investigate the underlying causes of severe hypokalemia once the patient has stabilized. Assess renal function, determine acid-base status, and any contributing factors that may lead to severe hypokalemia. Documentation of all interventions, reactions, and observations should be noted in the medical record. Arrange follow-up to prevent recurrences, while treating any root causes of hypokalemia. It is important to document effectively to maintain continuity of care for future reference and use [12].

In this patient, severe hypokalemia, elevated urine potassium levels, hypophosphatemia, severe glycosuria, and an elevated anion gap all pointed toward proximal convoluted tubule dysfunction, and a Fanconi-like syndrome was suspected as the most likely diagnosis. This case highlights the significance of taking a multidisciplinary approach in diagnosing electrolyte disorders including those involving potassium. Multidisciplinary approaches not only facilitate accurate diagnoses but also facilitate coordinated care throughout a patient's journey to promote better outcomes and quality of life. More research needs to be conducted to further elucidate the causes of proximal tubule dysfunction, including Fanconi syndrome, and develop therapies to improve longevity and quality of life in people living with these disorders.

## Conclusions

In this report, we highlighted the importance of diagnosis and management of hypokalemia, proximal convoluted tubule dysfunction, and a Fanconi-like syndrome in a patient who presented with cardiopulmonary arrest and severe, persistent hypokalemia. This case report illustrates the complexity and critical nature of diagnosing and managing hypokalemia in a patient with cardiac arrest. A comprehensive evaluation revealed proximal convoluted tubule dysfunction as a possible cause for her electrolyte imbalances and cardiac instability; the absence of common causes like medication adjustments or gastrointestinal symptoms necessitated further workup and multidisciplinary collaboration. Potassium supplementation and guideline-directed medical therapy for this patient's new heart failure ultimately led to improved outcomes, and she was discharged to a skilled nursing facility in a stable condition. More interdisciplinary research needs to be done to fully understand the complex interplay between proximal convoluted tubule dysfunction, hypokalemia, and cardiopulmonary events to pave the way for more diagnostic methods and therapeutic approaches to ultimately improve patient outcomes.
